# Autologous stem-cell transplantation in systemic sclerosis-associated interstitial lung disease: early action in selected patients rather than escalation therapy for all

**DOI:** 10.1177/1759720X211035196

**Published:** 2021-08-10

**Authors:** J. Spierings, Y-H. Chiu, M. Voortman, J. M. van Laar

**Affiliations:** Department of Rheumatology and Clinical Immunology, University Medical Centre Utrecht, Heidelberglaan 100, Utrecht, 3584 CX, the Netherlands; Division of Medicine, Department of Inflammation, Centre for Rheumatology and Connective Tissue Diseases, Royal Free and University College Medical School, University College London, London, UK; Department of Rheumatology and Clinical Immunology, University Medical Centre Utrecht, Utrecht, the Netherlands; Division of Rheumatology/Immunology/Allergy, Department of Medicine, Tri-Service General Hospital, National Defense Medical Center, Taipei; Department of Pulmonology, Division of Heart and Lungs, University Medical Centre Utrecht, Utrecht, the Netherlands; Department of Rheumatology and Clinical Immunology, University Medical Centre Utrecht, Utrecht, the Netherlands

**Keywords:** autologous stem cell transplantation, diffuse cutaneous systemic sclerosis, interstitial lung disease, review, scleroderma, systemic sclerosis

## Abstract

Systemic sclerosis (SSc) is a rare rheumatic disease characterised by inflammation, vasculopathy and fibrosis of skin and internal organs. A common complication and a leading cause of death in SSc is interstitial lung disease (ILD). The current armamentarium of treatments in SSc-ILD mainly includes immunosuppressive therapies and has recently been expanded with anti-fibrotic agent nintedanib. Autologous stem cell transplantation (SCT) is increasingly used in progressive diffuse cutaneous SSc. This intensive treatment has been studied in three randomised trials and demonstrated to improve survival and quality of life. In the subsets of patients with SSc-ILD, SCT resulted in stabilisation and modest improvement of lung volumes and disease extent on high resolution computed tomography, but less impact was seen on diffusion capacity. Comparison of SCT outcomes with results from SSc-ILD trials is difficult though, as lung involvement *per se* was not an inclusion criterion in all SCT trials. Also, baseline characteristics differed between studies. The risk of severe treatment-related complications from SCT is still considerable and patients with extensive lung disease are particularly at risk of complications during transplantation. Therefore SCT should only be provided by experienced multidisciplinary teams in carefully selected patients. Future research needs to include comprehensive pulmonary evaluation and establish whether SCT early in the disease might prevent irreversible pulmonary damage and reduce treatment-related complications. Also, more insight in mechanisms of action of SCT in the lung and predictors for response will improve the use of this treatment in SSc-ILD. In this review the role of SCT in the treatment of SSc-ILD is summarised.

## Introduction

Systemic sclerosis (SSc) is a rare connective tissue disease characterised by inflammation, vasculopathy and fibrosis of skin and internal organs.^1^ The clinical presentation of SSc is heterogenous and manifestations range from limited skin thickening to generalised skin involvement with severe internal organ damage. In the diffuse cutaneous disease subset (dcSSc) major organ involvement (heart, kidney and lungs) are common.^2^ Notably, pulmonary complications such as interstitial lung disease (ILD) compromise quality of life and are the leading cause of death in SSc.

In the last years the understanding of pathogenic pathways has improved. Damage to alveolar epithelial and endothelial cells leading to inflammation are regarded as the first central events in SSc-ILD.^3^ Ongoing damage and impaired healing of lung tissue together with aberrant innate and adaptive immune responses and myofibroblast function are believed to create a profibrotic milieu in the lung.^4,5^ Non-specific interstitial pneumonia is the most commonly observed radiological and histological pattern in SSc-ILD.^6^ Other patterns include usual interstitial pneumonia, organising pneumonia and diffuse alveolar damage.

Risk factors for development of SSc-ILD include dcSSc, shorter disease duration, male sex and older age at disease onset.^7,8^ Also the presence of anti-topoisomerase I antibodies has been identified as a predictor for SSc-ILD.^9^ The clinical course of SSc-ILD is variable as some patients have stable disease while others develop extensive and progressive disease.^10,11^ Therefore, pulmonary function tests (PFTs) and chest high resolution computed tomography (HRCT) play a central role in detection and follow-up of SSc-ILD.^12^

Current management options of SSc-ILD include immunosuppressive therapies and the recently approved anti-fibrotic agent nintedanib. In the case of refractory ILD, lung transplantation can be considered.^13^ Treatment recommendations and algorithms published over the years generally place mycophenolate mofetil (MMF) as the preferred first-line therapy and cyclophosphamide (CYC) and rituximab^14^ as second and third line, respectively.^15,16^ The place of autologous haematopoietic stem cell transplantation (SCT) in SSc-ILD has been a matter of debate. SCT has been shown to improve long-term event-free survival and overall survival in dcSSc patients, but the risk of treatment-related mortality restricts its use to a selection of patients. Notably, in the recently published European consensus statement on management of SSc-ILD, 80% of the expert panel agreed that SCT is a potential treatment in the case of rapid progressive and refractory lung disease.^17^ In this review we summarise the evidence on the effects of SCT on SSc-ILD and discuss the potential role of SCT in the treatment of SSc-ILD.

## Autologous SCT

SCT is an intensive immunomodulating therapy that has been used in the treatment of autoimmune diseases for more than 25 years.^18^ In the early years, SCT was mainly used to treat refractory cases with inflammatory arthritis.^19^ However, after the introduction of effective and less toxic biologic and other targeted agents, the role of SCT in the treatment of rheumatoid arthritis and juvenile idiopathic arthritis has diminished.^20,21^ In contrast, SCT is still performed in patients with Crohn’s disease, multiple sclerosis and SSc.^21^ In addition, recent reports on experiences with SCT in systemic lupus erythematosus, Behcet’s disease and vasculitis illustrate the need for this treatment in refractory cases of other rare autoimmune conditions.^22,23^

**Figure 1.**
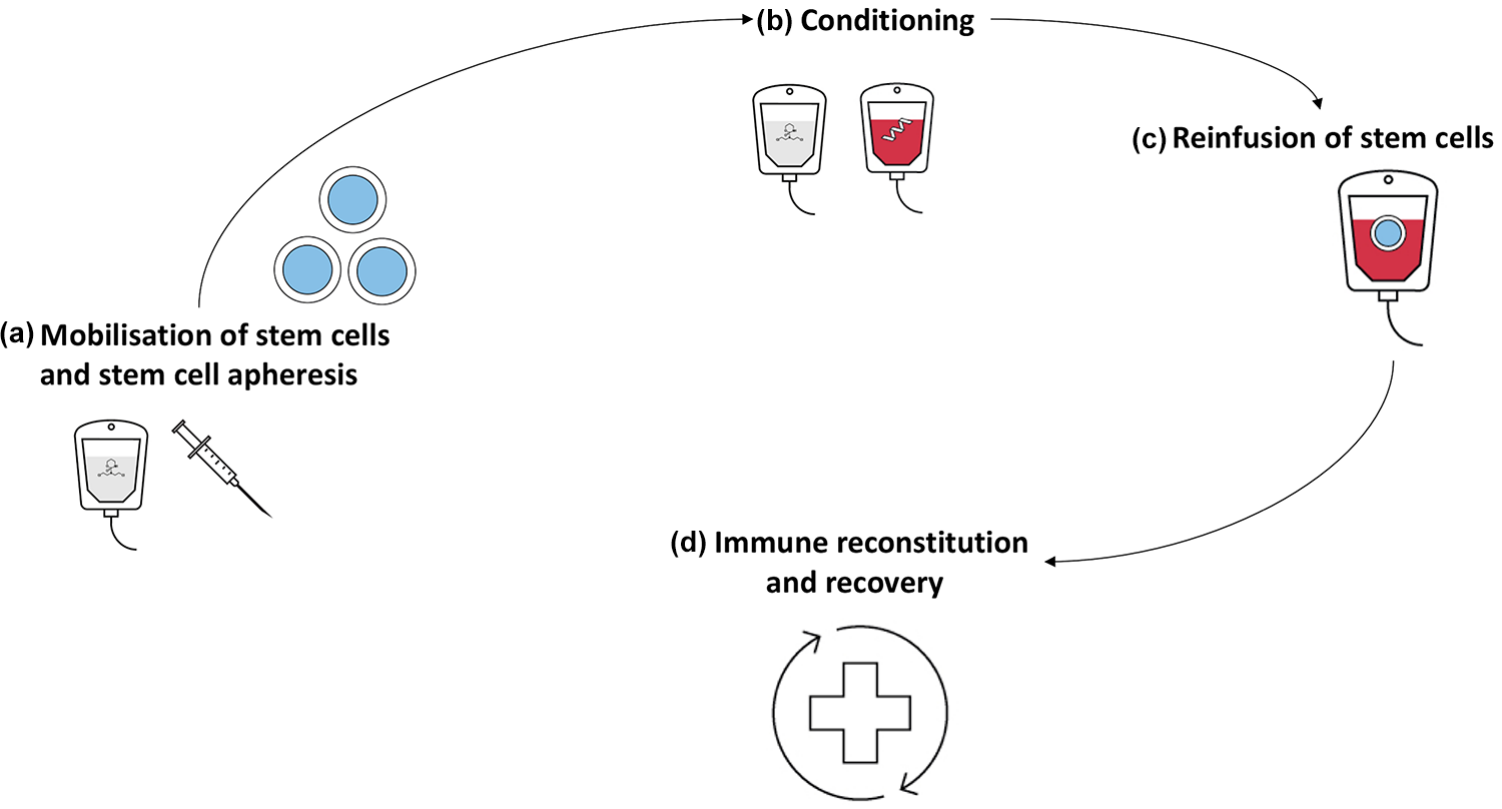
Autologous stem cell transplantation. (a) The first step in stem cell transplantation (SCT) is the mobilisation of stem cells from the bone marrow. This is most often done using chemotherapy, such as cyclophosphamide, to stimulate the production of stem cells in the bone marrow due to cytopenia. Granulocyte colony-stimulating factor is used to further facilitate the production and release of stem cells in the peripheral circulation. Subsequently, the stem cells are harvested using leukapheresis. (b) The next step is conditioning, which takes place approximately 4–6 weeks after mobilisation and leukapheresis. Myeloablative or highly immunosuppressive agents are administered, aiming to eliminate autoreactive B and T cells. Conditioning regimens in SCT for systemic sclerosis often include cyclophosphamide, anti-thymocyte globulin or total body irradiation. (c) Directly after completion of the conditioning scheme, stem cells are reinfused. Mostly graft manipulation is used (CD34+ selection), to improve efficacy of the treatment. (d) The last step involves supportive care during the aplastic phase, which normally takes 1 to 3 weeks until recovery. Full reconstitution of the immune system can take 6–9 months. Depending on the course of the treatment and condition of the patient pre-transplantation rehabilitation takes up several months.

SCT is thought to reset the immune system through elimination of autoreactive immune cells and regeneration of a new, rebalanced immune system. The exact mechanisms driving this reset are, however, not completely known.^24^ Autologous SCT consists of four steps (see Figure 1). The first step includes mobilisation of haematopoietic stem cells using chemotherapy (mostly CYC) and growth factors [granulocyte colony-stimulating factor (G-CSF)] to stimulate migration of stem cells from bone marrow to the blood so they can be collected using leukapheresis. This step is followed by conditioning which aims to eradicate autoreactive immune cells. Regimens used for conditioning can be either myeloablative or non-myeloablative and vary from high-intensive to intermediate-intensive schemes. In autoimmune diseases non-myeloablative intermediate intensive regimens are most commonly used. The third step is the reinfusion of autologous stem cells. *Ex vivo* graft selection (CD34+ selection) prior to reinfusion has been a matter of debate, although two studies recently demonstrated superiority of CD34+ selection compared with reinfusion of unselected cells in remission rate.^25,26^ The reinfusion of stem cells shortens aplasia from conditioning and allows a naïve immune system to emerge.

An important issue in SCT is the treatment-related mortality attributed to medication used for mobilisation and conditioning which can lead to severe infections, haemorrhage or cardiopulmonary toxicity. Therefore selection of patients, close monitoring during treatment and an experienced multidisciplinary team are key to ensure optimal and safe treatment. Also, benefits and risks need to be discussed with the patient carefully in order to make a balanced decision about treatment.^27^

## Impact of SCT on SSc-ILD

The benefits of SCT in progressive dcSSc on survival have been demonstrated in three controlled trials.^28–30^ In a meta-analysis a reduction of all-cause mortality compared with controls treated with CYC in progressive dcSSc [risk ratio (RR) 0.5 (95% confidence interval (CI), 0.33–0.75] was reported.^31^ Quality of life and skin involvement were also significantly better in patients treated with SCT. Although not all SSc patients had lung involvement in these trials and hence pulmonary endpoints were used as the sole primary outcome, the impact on lung disease is reported as co-primary or secondary outcome in all published trials and cohorts. Lung function parameters forced vital capacity (FVC) and diffusing capacity of the lungs for carbon monoxide (DLco) are most often reported and changes observed in HRCTs are described in a couple of studies. Change in pulmonary symptoms, patient reported outcomes or functional scores related to lung disease have not been reported yet.

### Impact of SCT on lung function

All three randomised controlled transplant trials in dcSSc report that SCT has beneficial effect on FVC but not significantly on DLco. The ASSIST study (American Scleroderma Stem Cell *versus* Immune Suppression Trial), which used >10% increase in FVC at 12 months as one of the two primary outcome measures, reported a significant improvement in FVC in the SCT group in 80% of patients (*n* = 8), while the mean FVC decreased in patients randomised to CYC (*n* = 9) one year post-transplantation.^28^ The mean rate of change of FVC in the SCT group was 10% in two years. Change in DLco did not differ significantly between groups. Four patients in the ASSIST trial had limited cutaneous SSc (lcSSc) with ILD. In the two patients treated with SCT lung function improved, whereas the two patients treated with cyclophosphamide pulse therapy experienced deterioration of pulmonary function.

The ASTIS trial (Autologous Stem Cell Transplantation International Scleroderma) observed a mean change in FVC of +6.3% at two years in the patients treated with SCT (*n* = 79) compared with −2.8% in the control arm (*n* = 77) (*p* = 0.004). A decrease of −4.7% in DLco in SCT-treated patients compared with −4.1% in the control group (*p* = 0.84) at 2 years of follow-up was seen.^29^ The SCOT trial (Scleroderma: Cyclophosphamide Or Transplantation) reported beneficial effects of SCT (*n* = 36) compared with cyclophosphamide (*n* = 39) on FVC but not on DLco.^30^ Fewer patients in the SCT group had a decrease of ⩾10% of FVC (*n* = 4) and more patients had improvement of FVC > 10% (*n* = 13), compared with the control group (*n* = 8 and *n* = 7, respectively) in the intention to treat analysis at 54 months. The majority of patients included in the ASTIS trial had mild ILD compared with patients in SCOT; this is reflected in the lung function results at baseline and should be taken into account when comparing these three trials.

Large observational studies reported similar findings for FVC and showed a modest positive effect on DLco as well. A retrospective analysis of transplant SSc patients in the Netherlands (*N* = 92, median follow-up time 4.6 years, 96% dcSSc, median disease duration 1.5 years, 36% had ILD) showed a median increase of FVC of +10% at 5 year follow-up and median increase of DLco from +6%. The Brazilian SCT cohort study (*N* = 70, median age 35.9, 57% female, 96% dcSSc, median disease duration at SCT was 2 years) reported stabilisation of both FVC and DLco after SCT.^32^ In patients with progressive ILD with decline in FVC or DLco > 10% in 6 months before SCT (*n* = 51), improvement of both FVC and DLco after treatment was observed at 5 year follow-up.

An analysis of the cohort of the European bone marrow transplant organisation (EBMT) (*N* = 80, per cent dcSSc not reported, follow-up time 2 years) reported an increase in FVC of +7% at 2 year follow-up (*p* < 0.001).^25^ DLco stabilised [+0.2% at 2 years (*p* = 0.01)]. A previous analysis in this cohort (*N* = 57, median age 40 years, 70% female, 88% dcSSc, median disease duration at SCT 36 months) showed no significant change in FVC or DLco during a follow-up period of 36 months, although serial long function parameters were available in only a small number of patients (*n* = 26 at 12 months, *n* = 18 at 24 months and *n* = 10 at 36 months) and 31% of patients had pulmonary arterial hypertension, which could influence lung function results too.^33^

A study by Nash *et al.*^34^ (*N* = 34, median age 41 years, 76% female, all dcSSc, median disease duration 21 months) followed patients for a median of 4 years (range 1–6) and reported a mean change in FVC from baseline to final evaluation of +2.1% (*p* = 0.50) and DLco of −6.0% (*p* = 0.05). Also, an observational Italian study (*N*= 18, median age 41 years, 72% female, all dcSSc, median disease duration at SCT 24 months) showed stabilisation of DLco at 60 month follow-up.^35^ (Table 1).

**Table 1.** SCT studies and effect on ILD.

Studies (N)	Regimen	Effect on lung function		Effect on HRCT
		FVC	DLco	
**ASSIST^28^** n = 10 (SCT) (70%*) n = 9 (CYC) (89%*) **Mean FU: 2.6** **years** **Primary outcome: improvement at** 12 months	**Mobilisation: CYC** 2 g/m^2^, G-CSF **Conditioning: CYC** (200 mg/kg), rabbit ATG **CD34 selection: no** **Comparator; CYC i.v. 6** **months**	**Baseline (median):** **SCT: 62% (**range **53–70)** **CYC: 67% (**range **43–84)** **Median change in 1 year:** **SCT: +20%** **CYC: −9%**	**Baseline (median):** **SCT:** 58% (range 29–82) **CYC: 75% (**range **29–111)** **Median change in 1 year:** **SCT: +9%** **CYC: −7%**	**Baseline (ILD on scan):** SCT: 70% CYC: 89% Change at 2 years: extent of ILD decreased after SCT but increased in controls
**ASTIS^29^** n = 79 (SCT) (86%*) n = 77 (CYC) (86%*) **Median FU: 5.8 years** **Primary outcome: EFS at 24 months**	**Mobilisation: CYC** 4 g/m^2^, G-CSF **Conditioning: CYC** (200 mg/kg), rabbit ATG **CD34 selection: yes** **Comparator; CYC i.v. 6** **months**	**Baseline (mean):** **SCT:** 82% (SD 19) **CYC:** 81% (SD 18) **Mean change in 2 years** **SCT: +6.3% (SD 18.3)** **CYC: −2.8 (SD 17.2)**	**Baseline (mean):** **SCT: 59% (SD 14)** **CYC: 58% (SD 14)** **Mean change in 2 years** **SCT: −4.7% (SD 13.7)** **CYC: −4.1 (SD 17.6)**	**Baseline (ILD on scan):** SCT: 87% CYC: 80%
**SCOT^30^** n= 36 (SCT) (100%*) n = 39 (CYC) (100%*) **Mean FU: 54** **months** **Primary outcome: GRCS** at 54 months	**Mobilisation:** G-CSF **Conditioning: CYC** (120 mg/kg), equine ATG TBI (800 cGy) **CD34 selection: yes** **Comparator; CYC i.v. 6** **months**	**Baseline (mean):** **SCT: 74**% (SD 15) **CYC: 74**% (SD 17) Change ITT group at 54 months: SCT *n* = 13 improvement** *n* = 10 no change *n* = 4 worsening** CYC *n* = 7 improvement *n* = 6 no change *n* = 8 worsening	**Baseline (mean):** **SCT: 54**% (SD 8) **CYC:** 53% (SD 8) Change ITT group at 54 months: **SCT** **n = 4** improvement*** **n = 19** no change **n = 13** worsening*** CYC *n* = 5 improvement *n* = 10 no change *n* = 24 worsening	**Baseline (ILD on scan):** SCT: 100% CYC: 95% Change at 54 months:**^36^** SCT Decreased ILD scores Stable fibrosis CYC No change ILD score Increased fibrosis
**Nash *et al.*^34^** N = 34 (79%*) Median follow-up 4 (range 1–6) years **Primary outcome: improvement of mRSS and HAQ-DI**	**Mobilisation: G-CSF** **Conditioning: TBI (800 cGY), CYC (120 mg/kg), and equine ATG (90 mg/kg)** **CD34 selection: yes** **Comparator; none**	**Baseline (median): 71 (range 27–103)** **Mean change in 4 years** **+2.1% [95% CI −5.2 to 9.3, (p = 0.560)]** **+1.7 per year (95% CI 0.4–3.0, p = 0.010)**	**Baseline (median): 62 (range 40–83)** **Mean change in 4 years** **−2.3% (95% CI −9.9 to 4.9, p = 0.310)** +0.4 per year (95% CI 1.4–0.7, *p* = 0.50)	Baseline HRCT (*n* = 34): Normal: 21% Ground-glass: 35% Fibrosis: 74% Change: 18%: ILD reactivation 18%: decreased ground-glass, increased fibrosis
**Bijnen *et al.*^37^** N = 92 (36%*) **Median FU: 5** **years (IQR 2–12 years)** **Primary outcome: EFS**	**Mobilisation: CYC 2–**4 g/m^2^, G-CSF **Conditioning: CYC** (200 mg/kg), rabbit ATG **CD34 selection: yes** **Comparator; none**	Baseline (median, *n* = 66): 84% (range 68–102%) Median at 5** **years (*n* = 40) 94% (range 81–107) +2.5 (1.9–3.0) per year	Baseline (median, *n* = 67): 55% (range 42–67%) Median at 5** **years (*n* = 38) 61% (range 53–73) +1.6 (1.0–2.2) per year	Median Goh scores Baseline (median, *n* = 39) 14% (range 7–34%) At 5** **years (median, *n* = 16) 8% (range 3–23%) −1.0 (−1.9 to 0.0) per year
**Henes *et al.*^25^** N = 80 (86%*) **FU: 2 years** **Primary outcome: PFS at 2** **years**	**Mobilisation: CYC 1–**4 g/m^2^, G-CSF **Conditioning: CYC** (50–240 mg/kg), rabbit ATG, thiotepa 10 mg/kg **CD34 selection: both** **Comparator; none**	Baseline (mean, *n* = 37) 74% (SD 16.9) Mean at 1** **year: 80% (SD 17) Mean at 2** **years: 81% (SD 19)	Baseline (mean, *n* = 35) 60% (SD 19.3) Mean at 1** **year: 60% (SD 18) Mean at 2** **year: 60% (SD 19)	–
**Henrique-Neto *et al.*^32^** N = 70 (84%*) **FU: 8** **years** **Primary outcome: –**	**Mobilisation: CYC 2** g/m^2^, G-CSF **Conditioning: 200** **mg/kg CYC and 4.5** **mg/kg ATG** **CD34 selection: yes** **Comparator; none**	Baseline (median, *n* = 70): 70 (range 35–122) *n* = 66 stabilisation Median at 5** **years (*n* = 51) 75% (range 48–110, *p* = 0.020)	Baseline (median, *n* = 70): 70 (range 48–125) *n* = 66 stabilisation Change at 5** **years (*n* = 51) 76% (range 50–115, *p* = 0.030)	–
**Farge *et al.*^33^** N = 57 (57%*) **FU: 36** **months**	**Mobilisation: CYC** 4 g/m^2^, +/−G-CSF **Conditioning: CYC** (150–200 mg/kg), other chemotherapy, rabbit ATG, TBI **CD34 selection: both** **Comparator; none**	Baseline (*n* = 47): 57% had FVC <70% **No significant change during 36** **months of FU**	Baseline (*n* = 47): 64% had DLco <70% **No significant change during 36** **months of FU**	
**Del Papa *et al.*^35^** N = 18 (67%*) **FU: 60** **months** **Primary outcome: –**	**Mobilisation: CYC** 4 g/m^2^, G-CSF **Conditioning: CYC** (200 mg/kg), rabbit ATG **CD34 selection: yes**		Baseline (median): 68% (range 51–100) **Median at 60** **months** **62% (range 30–85)**	–

* Percentage of patients with ILD.

** ⩾10%.

*** ⩾15%

ASSIST, American Scleroderma Stem Cell *versus* Immune Suppression Trial; ASTIS, Autologous Stem Cell Transplantation International Scleroderma; ATG, anti-thymocyte globulin; CI, confidence interval; CYC, cyclophosphamide; DLco, diffusing capacity of the lungs for carbon monoxide; EFS, event free survival; FU, follow-up; FVC, forced vital capacity; G-CSF, granulocyte colony-stimulating factor; GRCS, global rank composite scores; HAQ-DI, Health Assessment Questionnaire-Disability Index; HRCT, high resolution computed tomography; ILD, interstitial lung disease; IQR, interquartile range; ITT, intention-to-treat; i.v., intravenous; mRSS, modified Rodnan skin score; PFS, progression-free survival; SCOT, Scleroderma: Cyclophosphamide Or Transplantation; SCT, stem cell transplantation; SD, standard deviation; TBI, total body irradiation.

### Disease extent on imaging

Extent of ILD is generally assessed using thoracic HRCT scans and changes after SCT are described in only a couple of studies, which also use different outcome measures. In the retrospective Dutch cohort available HRCTs at baseline and 5 year follow-up were evaluated using Goh scores [a visual scoring (in per cent) of extent of SSc-ILD at HRCT].^38^ Median Goh scores improved from 14% (7–34%, *n* = 39) at baseline to 8% (3–23%, *n* = 16) at 5 year follow-up.^37^ Estimated mean improvement per year was −1.0 (95% CI −1.9 to 0.0). Another Dutch single-centre study evaluated HRCTs retrospectively at baseline and 1 year follow-up in 51 patients treated with either SCT (*n* = 20) or CYC (*n* = 31).^39^ A composite ILD score included assessment of total ILD extent, reticulations and ground glass opacities. Patients treated with SCT had clear improvement of ILD extent on HRCT at 1 year follow-up, and improved more (but not significantly) compared with the CYC-treated group [−5.1% of ILD score compared with −1.0% in the CYC group (*p* = 0.535) respectively]. Also, change in HRCT was weakly associated with change in PFT. Nash *et al.*^34^ evaluated HRCTs of 21 patients treated with SCT. The six patients who survived after 1 year follow-up had fewer ‘ground-glass’ abnormalities compared with baseline; however, more interstitial fibrosis was present compared with baseline.^28^ In the ASSIST trial the extent of lung disease decreased in patients treated with SCT at 2 year follow-up while this increased in controls.^28^

A German study used automated quantitative analysis on HRCTs of 26 patients (median age 41 years, 54% female, median disease duration 3.5 years) treated with SCT at 6 months and 2 years of follow-up.^40^ Based on FVC at 6 months patients were classified as responders (*n* = 20) and non-responders (*n* = 6). In these 20 responders DLco also significantly improved and total lung volume increased, lung density and high attenuation values decreased significantly. Additionally, structural and architectural properties of involved lung tissue parenchyma on chest computed tomography were analysed in 23 patients.^41^ Fibrotic features increased in non-responders (*n* = 5) at 6 and 12 months. In both responders (*n* = 18) and non-responders significant changes in these properties were observed at 6 months and at 12 months in responders only. A small French study (*N* = 9, median age 41 years, 67% female) qualitatively evaluated HRCTs at 6 up to 36 months and reported improvement on short term evaluation and stabilisation at the last follow-up scan.^42^ In conclusion, although different measures and scores were used in the studies and follow-up time was relatively short, most reported either stabilisation or improvement of lung disease on HRCT in patients treated successfully with SCT. In a sub-analysis of the SCOT trial, HRCTs were quantitatively scored on fibrosis and ILD scores during 54 months of follow-up.^36^ Patients treated with SCT showed decreased ILD scores and stable lung fibrosis compared with patients treated with CYC in the control arm.

### Progressive ILD or relapse after SCT

Approximately 17% of patients with SSc relapsed post-SCT.^20^ In the Dutch cohort study, 17 (18%) patients developed disease reactivation, mostly ILD (*n* = 11, 12%), requiring immunosuppressive medication.^32^ In the study by Nash *et al.*^34^ 29% (*n* = 6) experienced reactivation of lung disease after treatment. No data on newly developed ILD after SCT is described in the literature.

### Patient selection and pulmonary complications

A main concern in SCT is the risk of complications related to the treatment. Treatment-related mortality was considerably higher in dcSSc patients treated with SCT compared with control arms in the three randomised trials [RR 9.00 (95% CI, 1.57–51.69)].^31^ Patient selection for SCT therefore focuses on identifying patients at risk for SSc-related organ damage who are still in a fit state to undergo this intensive treatment without severe adverse events. This is reflected in the inclusion and exclusion criteria of trials (Table 2). It can be argued that the effect of SCT in patients with severe and active ILD might be larger compared with patients treated with mild ILD and therefore this needs to be taken into account while comparing results of different trials. Also, early SCT in patients with limited pulmonary disease may show less impact on present ILD in patients, but could prevent development or progression of ILD, which is currently being investigated in the UPSIDE trial.^43^

**Table 2.** Inclusion and lung-related exclusion criteria used in clinical trials.

Studies	Inclusion criteria	Lung-related exclusion criteria
ASSIST^28^	Age <60 years dcSSc Disease duration ⩽4 years mRSS ⩾15+ Internal organ involvement Lung: DLco <80% or FVC −10% within 12 months + HRCT abnormalities	TLC <45% of predicted PAH
ASTIS trial^29^	Age 18–65 years dcSSc Disease duration ⩽4years mRSS >15 Internal organ involvement Lung: DLco and/or FVC ⩽80% + HRCT abnormalities	DLco <40% of predicted PAH
SCOT^30^	Age 18–69 years dcSSc Disease duration ⩽4 years mRSS ⩾16 Internal organ involvement Lung: FVC <70% or DLco <70% + HRCT abnormalities	DLco <40% of predicted FVC <45% of predicted PAH
UPSIDE trial ^43^	Age 18–65 years dcSSc Disease duration ⩽2 years AND: mRSS ⩾15 OR: Internal organ involvement Lung: DLco and/or FVC ⩽85% and HRCT abnormalities or relative change in FVC >−10% or DLco >−15% within 12 months	DLco <40% of predicted PAH

ASSIST, American Scleroderma Stem Cell *versus* Immune Suppression Trial; ASTIS, Autologous Stem Cell Transplantation International Scleroderma; dcSSc, diffuse cutaneous systemic sclerosis; DLco, diffusing capacity of the lungs for carbon monoxide; FVC, forced vital capacity; HRCT, high resolution computed tomography; mRSS, modified Rodnan skin score; PAH, pulmonary arterial hypertension; SCOT, Scleroderma: Cyclophosphamide Or Transplantation; TLC, total lung capacity; UPSIDE, UPfront autologous haematopoietic Stem cell transplantation *versus* Immunosuppressive medication in early DiffusE cutaneous systemic sclerosis.

Severe pulmonary damage pre-treatment could place patients at risk of severe and even fatal treatment complications (an overview of pulmonary complications related to autologous SCT is provided in Table 3).^44^ In previous studies pulmonary complications were an important cause of death or organ failure after SCT. In the ASTIS trial, 15 (19%) severe pulmonary adverse events had occurred in the transplant group compared with six (7.8%) in the CYC arm.^29^ Fatal events included pulmonary haemorrhage, pulmonary oedema, acute respiratory distress syndrome (ARDS) triggered by G-CSF and pulmonary infection. In the SCOT trial, most events of organ failure were lung related as well.^30^ Five (13%) patients in the SCT arm died due to ARDS and pulmonary haemorrhage. In the recently published cohort studies fewer pulmonary complications were reported, which may be attributed to improved supportive care and increased awareness or possible underreporting. Thus, patient selection and collaboration with a multidisciplinary team including pulmonologists, infectious disease specialists and intensive care specialists is key to minimise risks for patients undergoing SCT.

**Table 3.** Pulmonary complications related to autologous stem cell transplantation.

Treatment phase	Complications
Mobilisation	
Pulmonary oedema	
G-CSF-related alveolitis	
Conditioning	
Pulmonary oedema	
ATG or cyclophosphamide toxicity	
Radiation-related lung damage	
Post-SCT	
Immune reconstitution inflammatory syndrome	
Haemorrhage	
Infection (bacterial, viral, fungal)	
Viral reactivation (CMV, EBV)	
TRALI after transfusion	

ATG, anti-thymocyte globulin; CMV, cytomegalovirus; EBV, Epstein–Barr virus; G-CSF, granulocyte colony-stimulating factor; SCT, stem cell transplantation; TRALI, transfusion-related acute lung injury.

## Mechanism of action of SCT

Immune reconstitution following SCT and the working mechanism of SCT have been studied in dcSSc and other autoimmune diseases, that is, multiple sclerosis and Crohn’s disease.^21^ Autoreactive immune cells and immune memory cells are erased, followed by reconstruction with CD34+ haematopoietic stem cells, which provide a chance to reshape by antigenic selection that may be different from the first triggering of diseases. Changes after SCT in both the innate immune system and the adaptive immune system have been described. In the SCOT trial normalisation of the interferon (IFN) signature, circulating neutrophils and NK cells was seen after treatment with SCT, but not in controls treated with CYC.^45^ Also, the diminished IFN and neutrophil gene signatures were associated with improved FVC. Other studies investigating reconstitution of innate immune responses reported changes in serum cytokine profiles after SCT, that is, IL-2 and IL-8, suggesting a shift in Th balance.^46–48^

The T cell receptor (TCR) repertoire showed up to 90% renewal two years after SCT.^49^ Broadening of the TCR repertoire is reflected by the increase in number of TCR-rearrangement excision circles and represents thymic output.^49^ Moreover, the Th1/Th2 ratio was found to increase after SCT at 1 month post-transplantation and reached a plateau after 6 months.^48^ B cell composition also changes following SCT and a decrease of IL-6- and TGF-β1 producing B cells and an increase of CD19^+^CD24^hi^CD38^hi^ B regulatory cells (Bregs) were observed after treatment in 22 patients.^50^ Interestingly, the number of CD19^+^CD24^hi^CD38^hi^ Bregs at baseline was also associated with post-SCT remission. In another study (*N* = 17) decline in both naïve and memory B cells was seen until one year post-transplantation and lower peripheral B cell levels were associated with infectious complications.^51^

Although more insight has been gained in the reconstitution of circulation immune cells, it is still unclear how SCT induces immunological changes in peripheral tissue, including the lungs. As illustrated by the varied clinical response reported, improvement is much more prominent in skin compared with lungs or the gastrointestinal tract; SCT may impact pathogenic processes in every organ differently.^34^ Although there is limited evidence on predictors for pulmonary outcome after SCT, several biomarkers are correlated with clinical response in studies investigating other treatments for SSc-ILD. For instance, decrease in serum level of Krebs von den Lungen 6 and surfactant protein D are associated with improvement of FVC after SCT,^48^ and changes in bronchoalveolar lavage proteins have shown to predict treatment response.^52^

## Implications for further research

With three randomised controlled clinical trials completed and countries sharing their experiences with SCT with cohort studies, understanding of the effects of SCT on organ complications such as ILD has grown. Currently the UPSIDE trial is ongoing and investigates upfront SCT in early disease compared with other immunosuppressive therapy and the impact on ILD, using lung function and imaging (automated quantitative HRCT analysis and positron emission tomography) scans to assess changes in the lung after treatment.^43^ Also the use of post-transplant MMF in order to prevent (pulmonary) relapse is currently under investigation (NCT01413100). Still, no studies have been done investigating the impact of SCT compared with immunosuppressive medication in the long-term using lung involvement as a primary outcome measure. Future research focused on lung involvement is therefore needed. Additionally, studies are required to investigate refined treatment strategies with similar or better effects but lower toxicity making SCT suitable for patients with more extensive disease who are currently excluded for this treatment. Also the impact of SCT in patients with lcSSc-ILD has yet to be established as only very few cases with lcSSc-ILD treated with SCT were included in the studies and outcomes in this subset are described only in the ASSIST trial. Furthermore, as mentioned above, not much is known about the impact of SCT on lung-related patient reported outcomes. Mechanistic studies investigating changes in the lungs during and after SCT could improve understanding of the different effect of the treatment on ILD compared with skin fibrosis and might help to identify biomarkers predicting response to SCT or immunosuppressive treatment in early onset.

## Discussion

In this review we summarised the results of SCT on SSc-ILD. Autologous SCT showed a modest but clinically relevant improvement of lung volumes and disease extent on imaging; however, no consistent effect on DLco has been reported. This small effect on DLco may be explained by coexisting pulmonary vascular disease which is less affected by SCT.^53^ Moreover, other factors can influence FVC, such as myositis or other chest problems, or affect DLco, including anaemia, intrapulmonary or intracardiac shunts and cardiac disease.^54^ That DLco results can be affected by cardiac involvement was also shown in a retrospective analysis of 90 SSc patients treated with SCT.^55^ In this study DLco did not improve significantly after treatment in the whole group, but only in patients with normal cardiac tests (echo and electrocardiogram) at baseline. Thorough pre-transplant screening in microvascular and cardiac disease is therefore essential not only for risk assessment during the treatment but also to anticipate response.

Robust evidence for the efficacy of SCT in SSc-ILD is, however, still lacking as none of the controlled SCT studies was primarily powered for lung outcomes. Comparison between SCT studies and trials investigating immunosuppressive therapies in SSc-ILD is also limited due to differences in inclusion criteria and subsequently baseline characteristics, treatment regimens and clinical endpoints. Importantly, ILD was not a sole inclusion criterion in the SCT trials so as a consequence not all included patients in these studies had ILD at baseline, while in studies investigating the impact of immunosuppressive and antifibrotic therapies all participants had established SSc-ILD. Furthermore, no outcome measures on impact on symptoms and pulmonary performance in daily life for patients are collected in published SCT trials.^56^

Currently, MMF is the first treatment choice for SSc-ILD as it has a favourable safety profile and was demonstrated to stabilise lung function after two years in the Scleroderma Lung study II.^57^ Also, CYC still has a place in the treatment of SSc-ILD, when followed by other disease-modifying anti-rheumatic drug therapies.^58–60^ Biologics such as rituximab have demonstrated benefits in SSc-ILD by improving both restriction and diffusion capacity in a meta-analysis,^14^ and subcutaneous tocilizumab showed a trend towards stabilisation of FVC.^61,62^ Particularly, tocilizumab seems to stabilise lung function decline in patients with early SSc-ILD and elevated acute-phase reactants,^63^ and in patients with positive anti-topoisomerase antibodies.^64^ Nintedanib managed to slow down FVC decline, and can be a potential addition to immunosuppressive therapies such as MMF.^65,66^

Although new immunomodulating and combined treatment with antifibrotic therapies are emerging into the clinics and will be first-line therapy for most patients with SSc-ILD, SCT remains a potent treatment that could prevent progression of SSc-ILD on the long-term in patients with early rapidly progressive dcSSc. International guidelines recommend SCT in a careful selection of SSc patients in highly experienced centres.^67^ Accordingly, the recent European consensus guidelines adopted SCT as an escalation treatment for a subset of patients with SSc-ILD.^17^ Unfortunately, details about this selected subset that could guide treatment decisions are not mentioned in this guideline. Based on the existing literature there is only evidence for SCT in dcSSc-ILD patients as lcSSc-ILD patients were not included in most studies. Furthermore, SCT trials included patients with rapidly progressive and early disease rather than refractory cases, as is suggested by the European consensus guideline. We therefore recommend that SCT is used in line with eligibility criteria of the ASTIS and SCOT trials only in dcSSc-ILD patients. Caution should be taken in patients with extensive, refractory ILD because of the risk of (pulmonary) complications related to SCT procedures and infections as described in this review, and the lack of evidence of efficacy of SCT in this group of patients. Future research is needed to refine treatment strategies in patients with lcSSc-ILD and patients with extensive disease and subsequent high risk of complications, to establish impact of SCT on patient-reported outcomes and identification of predictors for response. Also, the ongoing UPSIDE trial may shed light on the impact of upfront SCT on SSc-ILD as this trial also evaluates lung outcomes measures comprehensively.

In conclusion, autologous SCT in dcSSc is a powerful treatment option which can stabilise and even improve lung involvement in a selected group of patients with dcSSc; however, more research is needed to further determine its role in the management of SSc-ILD.
